# Dosimetric analysis of local failures in skull-base chordoma and chondrosarcoma following pencil beam scanning proton therapy

**DOI:** 10.1186/s13014-020-01711-3

**Published:** 2020-11-16

**Authors:** Lucas Basler, Robert Poel, Christina Schröder, Alessandra Bolsi, Antony Lomax, Stephanie Tanadini-Lang, Matthias Guckenberger, Damien C. Weber

**Affiliations:** 1 Center for Proton Therapy, Paul Scherrer Institute, ETH Domain, Forschungsstrasse 111, 5232 Villigen, Switzerland; 2 Department of Radiation Oncology, University Hospital Zürich, University of Zurich, Zurich, Switzerland; 3 Department of Radiation Oncology, University Hospital Bern, University of Bern, Bern, Switzerland

**Keywords:** Chordoma, Chondrosarcoma, Skull-base, Pencil beam scanning proton therapy, Proton therapy, Patterns of recurrence, Dosimetric analysis

## Abstract

**Background:**

Despite combined modality treatment involving surgery and radiotherapy, a relevant proportion of skull-base chordoma and chondrosarcoma patients develop a local recurrence (LR). This study aims to analyze patterns of recurrence and correlate LR with a detailed dosimetric analysis.

**Methods:**

222 patients were treated with proton radiotherapy for chordoma (n = 151) and chondrosarcoma (n = 71) at the PSI between 1998 and 2012. All patients underwent surgery, followed by pencil-beam scanning proton therapy to a mean dose of 72.5 ± 2.2Gy_RBE_. A retrospective patterns of recurrence analysis was performed: LR were contoured on follow-up MRI, registered with planning-imaging and the overlap with initial target structures (GTV, PTV_high-dose_, PTV_low-dose_) was calculated. DVH parameters of planning structures and recurrences were calculated and correlated with LR using univariate and multivariate cox regression.

**Results:**

After a median follow-up of 50 months, 35 (16%) LR were observed. Follow-up MRI imaging was available for 27 (77%) of these recurring patients. Only one (3.7%) recurrence was located completely outside the initial PTV (surgical pathway recurrence). The mean proportions of LR covered by the initial target structures were 48% (range 0–86%) for the GTV, 70% (range 0–100%) for PTV_high_ and 83% (range 0–100%) for PTV_low_. In the univariate analysis, the following DVH parameters were significantly associated with LR: GTV(V < 66Gy_RBE_, *p* = 0.01), GTV(volume, *p* = 0.02), PTV_high_(max, *p* = 0.02), PTV_high_(V < 66Gy_RBE_, *p* = 0.03), PTV_high_(V < 59Gy_RBE_, *p* = 0.02), PTV_high_(volume, *p* = 0.01) and GTV(D95, *p* = 0.05). In the multivariate analysis, only histology (chordoma vs. chondrosarcoma, *p* = 0.01), PTV_high_(volume, *p* = 0.05) and GTV(V < 66Gy_RBE_, *p* = 0.02) were independent prognostic factors for LR.

**Conclusion:**

This study identified DVH parameters, which are associated with the risk of local recurrence after proton therapy using pencil-beam scanning for patients with skull-base chordoma and chondrosarcoma.

## Background

Chordoma and chondrosarcoma of the skull base are rare bone tumors [1–4] which are often located in close proximity to critical organs of risk (OAR), including but not limited to the brainstem, optic chiasm and optic nerves, making complete surgical resection of these tumors challenging and postoperative high-dose radiotherapy necessary in most cases [5].

Particle beam radiotherapy offers critical advantages compared to photon irradiation in these patients [6, 7], not only limited to protons but also carbon ions, as both are able to deliver high curative doses even to target volumes in close proximity to OARs, based on their finite range in tissue (Bragg-Peak), as well as an overall lower integral radiation dose delivered to the patient.

However, despite combined modality treatment involving surgery and adjuvant radiotherapy, a relevant percentage of chordoma and chondrosarcoma patients develop a local recurrence [8–10], which is most likely explained by the locally aggressive growth pattern. Unfortunately, in most cases, we currently lack prognostic parameters to assess the risk of local failure of an individual patient. Identifying patient, tumor or treatment characteristics, associated with an increased risk of local failure, could help in the clinical decision-making process.

In a previous study, we identified optic apparatus and/or brainstem compression, histology and GTV volume as prognostic factors for the risk of local failure [11]. The present study aims to analyze patterns of recurrence and correlate local control with a detailed dosimetric analysis.

## Methods

### Patient characteristics

This study is based on overall 222 patients, which were treated with proton radiotherapy for chordoma (n = 151, 68%) or low-grade chondrosarcoma (71, 32%) at the Paul Scherrer Institute (PSI) between 1998 and 2012. Written informed consent was obtained from all patients and the study was approved by the local ethics committee (Kantonale Ethikkommission Nordwest- und Zentralschweiz (EKNZ, approval number 2020-01071) in accordance with ‘good clinical practice’ (GCP) guidelines and the Declaration of Helsinki. The patient’s characteristics are detailed in Table 1 and have also been described in our previous analysis [11].

**Table 1:** Patient characteristics of 222 patients treated with pencil-beam scanning proton therapy

Parameter	Total *N* = 222	Chordoma *N* = 151	ChSa *N* = 71	*p* value
*Age (years)*				
Mean (SD)	40.8 (18.4)	43.3 (18.1)	35.6 (18.3)	*0.004*
*Gender*				
Female (%)	105 (47.3)	65 (43.0)	40 (56.3)	0.09
Male (%)	117 (52.7)	86 (57.0)	31 (43.7)	
*Recurrent disease*				
No (%)	171 (77)	115 (76.2)	56 (78.9)	0.76
Yes (%)	51 (23)	36 (23.8)	15 (21.1)	
*GTV (cc)*				
Mean (SD)	35.7 (29.1)	35.4 (27.5)	36.1 (32.5)	0.87
*Brainstem compression*				
No (%)	151 (68)	100 (66.2)	51 (71.8)	0.62
Abutment (%)	46 (20.7)	34 (22.5)	12 (16.9)	
Yes (%)	25 (11.3)	17 (11.3)	8 (11.3)	
*Optic apparatus compression*				
No (%)	154 (69.4)	113 (74.8)	41 (57.7)	*0.03*
Abutment (%)	44 (19.8)	26 (17.2)	18 (25.4)	
Yes (%)	24 (10.8)	12 (7.9)	12 (16.9)	
*Any compression*				
No (%)	109 (49.1)	76 (50.3)	33 (46.5)	0.7
Yes (%)	113 (50.9)	75 (49.7)	38 (53.5)	
*Surgery*				
Subtotal resection (%)	215 (96.8)	147 (97.3)	68 (95.8)	0.68
Complete resection (%)	7 (3.2)	4 (2.7)	3 (4.2)	
*Number of surgeries*				
1 (%)	101 (46.8)	63 (43.4)	38 (53.5)	*0.013*
2 (%)	78 (36.1)	59 (40.7)	19 (26.8)	
3 (%)	23 (12.0)	14 (9.7)	12 (16.9)	
4 (%)	6 (2.8)	6 (4.1)	0 (0)	
5 (%)	3 (1.4)	3 (2.1)	0 (0)	
6 (%)	2 (0.9)	0 (0)	2 (2.8)	
*Postoperative complications*				
No (%)	154 (69.4)	106 (70.2)	48 (67.6)	0.89
Yes (%)	68 (30.6)	45 (29.8)	23 (32.4)	

Italics indicate statistically significant differences

### Patient treatments

All patients underwent surgery with 215 subtotal/near complete resections (96.8%) and 7 complete resections (3.2%). The gross tumor volume (GTV) was based on postoperative planning CT and MRI imaging and had a mean volume of 35.7 ± 29.1 cm^3^. The clinical target volume (CTV) was based on the preoperative tumor extension including regions of suspected microscopic spread, including the surgical pathway based on institutional guidelines. The patients were treated with a larger (GTV or CTV expansion of 10 mm in most cases) low-dose PTV_low_ to a dose of 54 Gy_RBE_ and received a sequential boost to a smaller (GTV or CTV expansion of 5 mm in most cases) high-dose PTV_high_ to a dose of 74 Gy_RBE_ or 70 Gy_RBE_ for chordoma and chondrosarcoma respectively. Proton therapy was administered using pencil-beam scanning (PBS) technique to a mean dose of 72.5 ± 2.2 with a prescribed dose of 74 Gy_RBE_ for chordoma and 70 Gy_RBE_ for chondrosarcoma patients at 1.8–2.0 Gy_RBE_ per fraction. Relative biologic effectiveness (RBE) was defined as 1.1 [12]. GTV coverage was maximized with regard to OAR dose constraints. Patients were treated exclusively with single-field uniform dose (SFUD) until 2003 and afterwards with a combination of intensity modulated proton therapy (IMPT). The first treatment series was typically delivered with three- or four-field SFUD plans, including one or two opposed lateral fields with a small couch kick and two superior oblique fields. IMPT plans were optimized with four non-coplanar fields, two posterior oblique and two anterior oblique fields. Additional information regarding irradiation technique, planning objectives and OAR dose constraints, has been previously published [13].

### Follow-up evaluation

Follow-up was performed in intervals of 3–6 months for the first 2–3 years and annually thereafter via clinical assessment and MRI, as well as CT imaging. Local control was defined as a reduction in tumor volume or stable disease comparing follow-up MRI and CT images with imaging prior proton therapy.

### Patterns of recurrence and DVH analysis

**Fig. 1:**
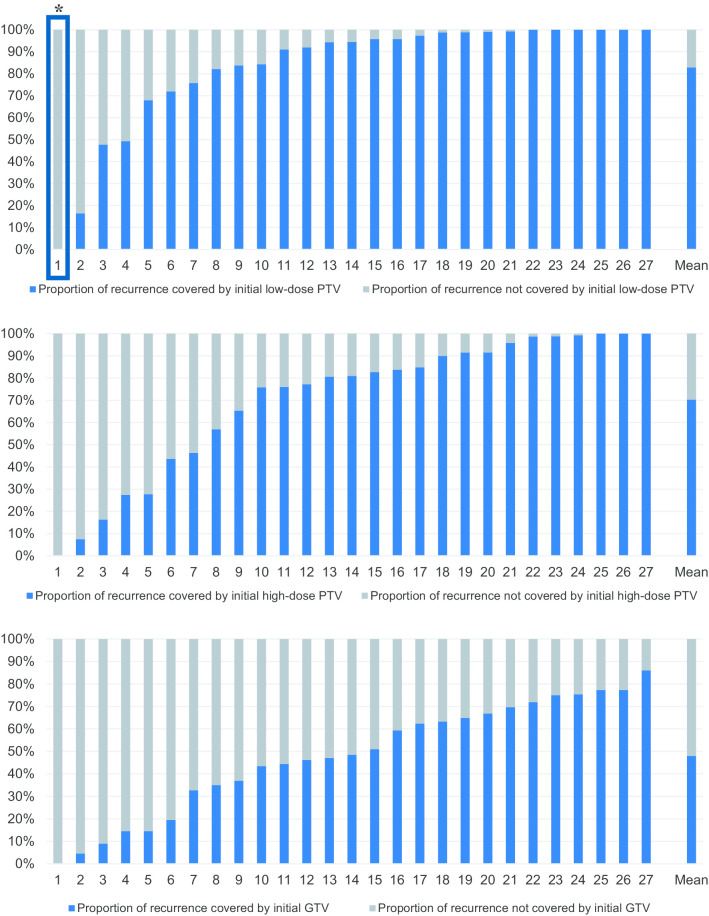
Proportions of the recurrence covered or not covered by the initial target structures (low-dose PTV, high-dose PTV, GTV). Only one recurrence was located completely outside the initial PTV (patient 1) and was located within the surgical pathway. The mean proportions of the recurrence covered by the initial target structures were 48% (0–86%) for the GTV, 70% (0–100%) for the high-dose PTV_high_ and 83% (0–100%) for the low-dose PTV_low_. *Surgical pathway recurrence

An in-depth retrospective patterns of recurrence and dose volume histogram (DVH) analysis was performed. The initial target contour sets were exported from our in-house treatment planning system (PSIplan) into the treatment planning software Eclipse (Version 13.6, Varian Medical Systems, Palo Alto, USA) and used for both the patterns of recurrence and DVH analysis. The local recurrences were contoured on the respective follow-up MRI imaging studies, registered with the initial planning CT and MRI images and the newly generated structures (i.e. recurrences) were also exported into Eclipse to calculate the overlap of the local failures with the initial target structures GTV, PTV_high_ and PTV_low_ (Fig. 1). In addition, individual DVH parameters of the initial planning structures were calculated for all 222 patients. The DVH parameters of patients with locally controlled tumors were statistically compared to the available DVH parameters of the patients who developed a local recurrence (n = 32 patients).

### Statistical analysis

Local control (LC) was assessed, comparing patients with and without recurrence, as well as with and without OAR involvement. Local control was defined as stable disease or tumor shrinkage, while radiologically confirmed tumor growth in subsequent images was considered a local failure/recurrence. In our previous study, we compared the prognostic potential of clinical (histology, patient’s age, gender, compression of the brainstem or optic apparatus, tumor volume, recurrent status before PT) and technical (number of surgeries, weekly fractions) factors for both local control and overall survival (OS) [11]. In the present study, individual DVH parameters including D_min_, D_max_, D98, D95, D_mean_, V70, the volume receiving less than 66 Gy_RBE_ (V < 66Gy_RBE_), and the volume receiving less than 59 Gy_RBE_ (V < 59Gy_RBE_) of initial planning structures and recurrences were calculated. These parameters were statistically compared between patients with and without locally controlled tumors to derive prognostic DVH parameters for LC via univariate and multivariate cox regression. To account for the outcome differences between skull base tumor histology, this was included as a categorial variable in the multivariate analysis. Concordance index (CI) was calculated for all Cox models. Statistical analysis was performed using R software. Tests were performed two-sided and differences with a *p* value below 0.05 were considered as statistically significant.

## Results

After a median follow-up of 50 months (range 4–176), 35 (16%) local failures were observed, 5 (7%) in chondrosarcoma and 30 (20%) in chordoma patients. As described in our previous analysis, the 5- and 7-year local control rates were 81% and 78% (all patients), 76% and 71% (chordoma) and 94% (chondrosarcoma), respectively. Local control for chondrosarcomas was significantly better compared to chordomas (*p* = 0.04, CI = 0.59). 5- and 7-year overall survival for the entire cohort were 86% and 80%, respectively [11].

Follow-up MRI imaging, which was the basis for diagnosis of local recurrence, was available for 27 (77%) of these 35 recurring patients. The initial PTV including DVH could be extracted for 213 (96%) of the total studied cohort and for 32 (91%) of the recurring patients. In some patients, only an initial CTV was available which led to a slight decrease in available GTV structures of 194 (87%) for the total cohort. For the recurring patients, there was no difference and the initial GTV was available for 32 (91%) patients. Thus, the dosimetric evaluation was based on the exported DVH data of 32 patients, while the patterns of recurrence analysis was performed on 27 patients where follow-up imaging was available. It is important to note that neither target delineation principles, nor treatment technique or planning objectives differed between recurring and non-recurring patients.

The overlap of the local failures with the initial target structures GTV, high-dose PTV_high_ and low-dose PTV_low_ was calculated. Only one recurrence was located completely outside the initial PTV (Fig. 1, patient 1) and was identified as a surgical pathway recurrence. The mean proportion of recurrence volumes covered by the initial target structures (overlap) were 48% for the initial GTV, ranging from 0% (surgical pathway recurrence) to 86% (local failure). The initial PTV_high_ covered a mean of 70% of the recurrence volumes, ranging from 0 to 100%. The larger PTV_low_ covered a mean of 83% of recurrence volumes, ranging from 0 to 100% (Fig. 1).

**Fig. 2:**
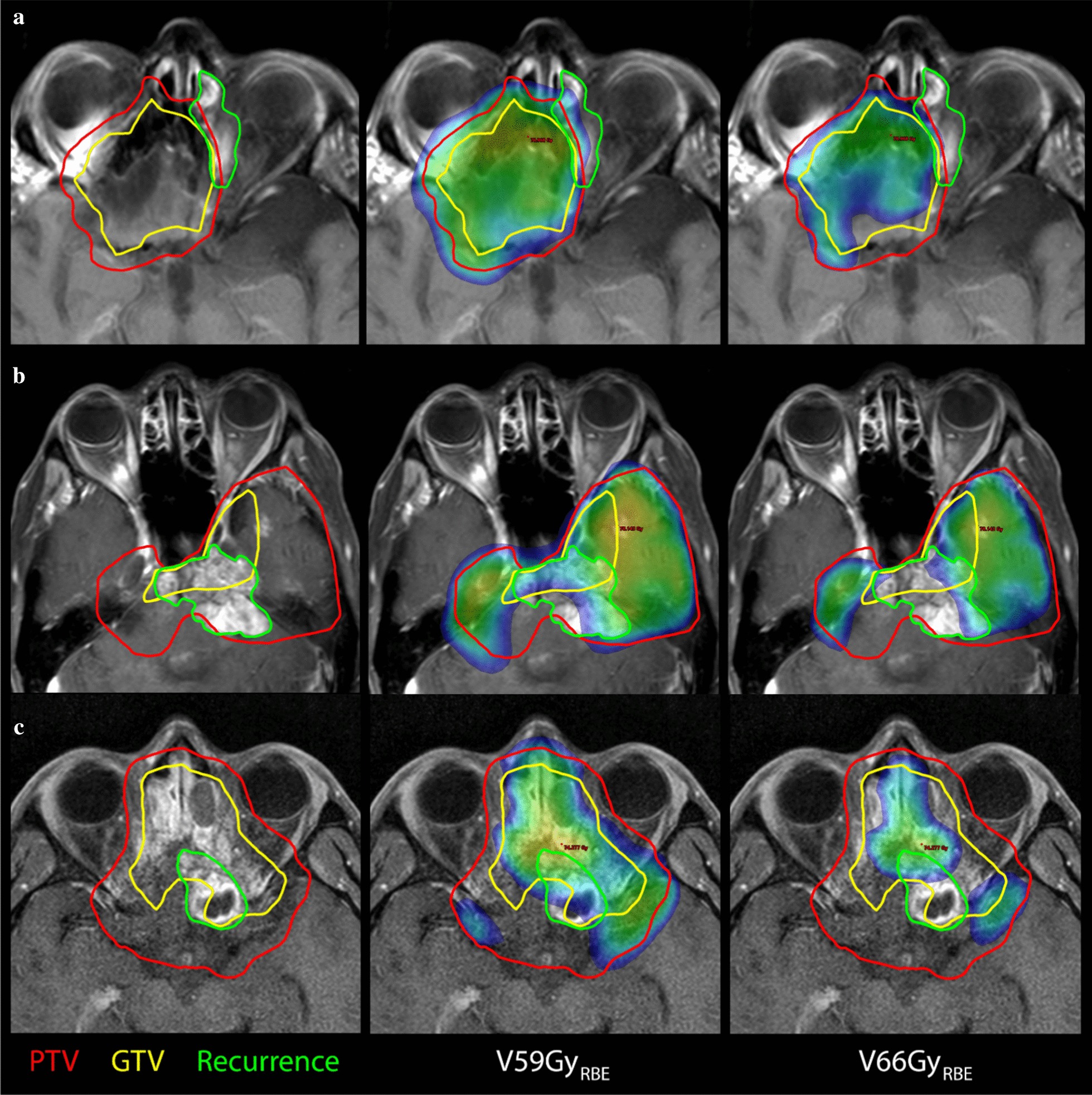
Three examples of in-field recurrences (green) or marginal failures, together with the initial GTV (yellow) and PTV_high_ (red) structures and dose distribution showing V59Gy_RBE_ and V66Gy_RBE_ (in color wash). The dose of 66 Gy_RBE_ corresponds to the 95% covering isodose (D95) in most cases. The first example (**a**) shows a marginal failure in the ethmoid/frontal sinus due to lack of elective coverage in this area. In the second example (**b**), the initial PTV_high_ (red) nearly completely covers the recurrence (green) but because of the brainstem OAR constraint, the dose distribution is compromised in this area (right image). Example 3 (**c**) shows a marginal failure because of chiasm OAR constraints

Three examples of in-field recurrences or marginal failures, together with the initial GTV (yellow) and PTV_high_ (red) structures and dose distribution showing V59Gy_RBE_ and V66Gy_RBE_ (in color wash) are shown in Fig. 2. The dose of 66 Gy_RBE_ corresponds to 95% of the prescription dose (D95) in most cases. The first example (A) shows a marginal failure in the ethmoid/frontal sinus, which may be due to lack of elective coverage in this area. In the second example (B), the initial PTV_high_ (red) almost completely covers the recurrence (green) but because of the brainstem OAR constraint, the dose distribution is compromised in this area (right image). Example 3 (bottom) shows a marginal failure, likely because of chiasm OAR constraints.

**Fig. 3:**
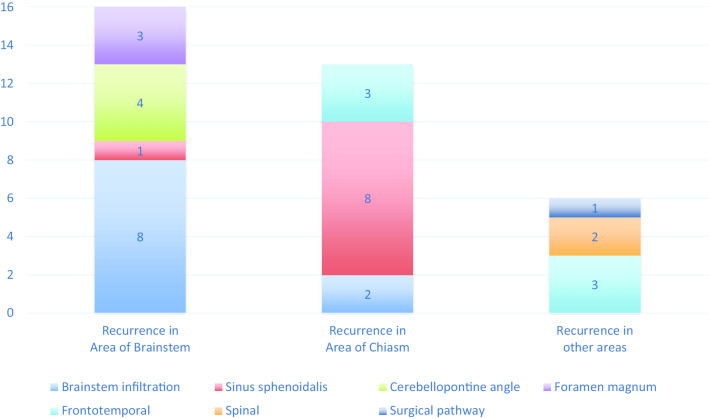
Patterns of recurrence by location. Two of the recurred patients (6%) had a recurrence in both the area of brainstem and chiasm (not shown). Recurrences that occurred in the area of the brainstem (n = 16, 46%) had a tendency to compress or infiltrate the brainstem (n = 8, 23%) or occurred in the cerebellopontine angle (n = 4, 11%) or the foramen magnum (n = 3, 9%), while sphenoid sinus infiltration was rare (n = 1, 3%). Recurrences occurring in the area of the chiasm (n = 13, 37%) were highly likely to infiltrate the sphenoid sinus (n = 8, 23%) with only a small percentage of brainstem infiltration (n = 2, 6%) or progression to the frontotemporal brain tissue (n = 3, 9%). There were 6 recurrences (17%) outside the brainstem or chiasm areas, 3 (9%) in the frontotemporal brain, 2 (6%) in the spinal area and one (3%) surgical pathway recurrence in the skull

The location of the recurrence was analyzed and categorized into three groups (brainstem, chiasm, other, Fig. 3). Recurrences that occurred in the area of the brainstem (n = 16, 46%) had a tendency to compress or infiltrate the brainstem (n = 8, 23%) or occurred in the cerebellopontine angle (n = 4, 11%) or the foramen magnum (n = 3, 9%). Sphenoid sinus infiltration was rare (n = 1, 3%). Recurrences occurring in the area of the chiasm (n = 13, 37%) infiltrated the sphenoid sinus (n = 8, 23%) most frequently with only a small percentage of brainstem infiltration (n = 2, 6%) or progression to the frontotemporal brain tissue (n = 3, 9%). Two of the recurred patients (6%) had a recurrence in both the area of brainstem and chiasm. There were 6 recurrences (17%) outside the brainstem or chiasm areas, 3 (9%) in the frontotemporal brain, 2 (6%) in the spinal area and one (3%) surgical pathway recurrence in the skull.

In addition to the patterns of recurrence analysis, individual DVH parameters of initial planning structures and recurrences were calculated and correlated with local failure (Table 2). In the univariate analysis, we identified several conventional clinical DVH parameters, which were significantly associated with an increased risk of local recurrence. For the GTV the parameters were: GTV (D95, *p* = 0.05), GTV volume receiving less than 66 Gy_RBE_ (V < 66Gy_RBE_, *p* = 0.01), GTV volume (*p* = 0.02). For the PTV_high_ significant factors include: PTV_high_ (D_max_, *p* = 0.02), PTV_high_ volume receiving less than 66 Gy_RBE_ (V < 66Gy_RBE_, *p* = 0.03), PTV_high_ volume receiving less than 59 Gy_RBE_ (V < 59Gy_RBE_, *p* = 0.02), PTV_high_ volume (*p* = 0.01).

**Table 2:** Prognostic DVH parameters

DVH parameters and histology		Univariate analysis		Multivariate analysis	
		*p* value	CI	*p* value	CI
GTV	D95 (%)	*0.05*	0.59	ns	ns
GTV	V < 66 Gy_RBE_	*0.01*	0.62	*0.022*	0.67
GTV	Volume	*0.02*	0.64	ns	ns
PTV_high_	Max dose Gy_RBE_	*0.02*	0.61	ns	ns
PTV_high_	V < 66 Gy_RBE_	*0.03*	0.63	ns	ns
PTV_high_	V < 59 Gy_RBE_	*0.02*	0.62	ns	ns
PTV_high_	Volume	*0.01*	0.63	*0.045*	0.67
Histology	Chordoma versus chondrosarcoma	–	–	*0.009*	0.67

Italics indicate statistically significant differences

In the univariate analysis, the following DVH parameters were significantly associated with an increased risk of local recurrence: GTV D95 (*p* = 0.05), GTV volume receiving less than 66 Gy_RBE_ (V < 66Gy_RBE_, *p* = 0.01), GTV volume (*p* = 0.02), PTV maximum dose (*p* = 0.02), PTV volume receiving less than 66 Gy_RBE_ (V < 66Gy_RBE_, *p* = 0.03), PTV volume receiving less than 59 Gy_RBE_ (V < 59Gy_RBE_, *p* = 0.02), PTV volume (*p* = 0.01). In the multivariate analysis, histology (chordoma vs chondosarcoma, *p* = 0.009), PTV volume (*p* = 0.045) and GTV (V < 66GyRBE, *p* = 0.022), (CI = 0.67) presented as independent prognostic factors of local tumor control

**Fig. 4:**
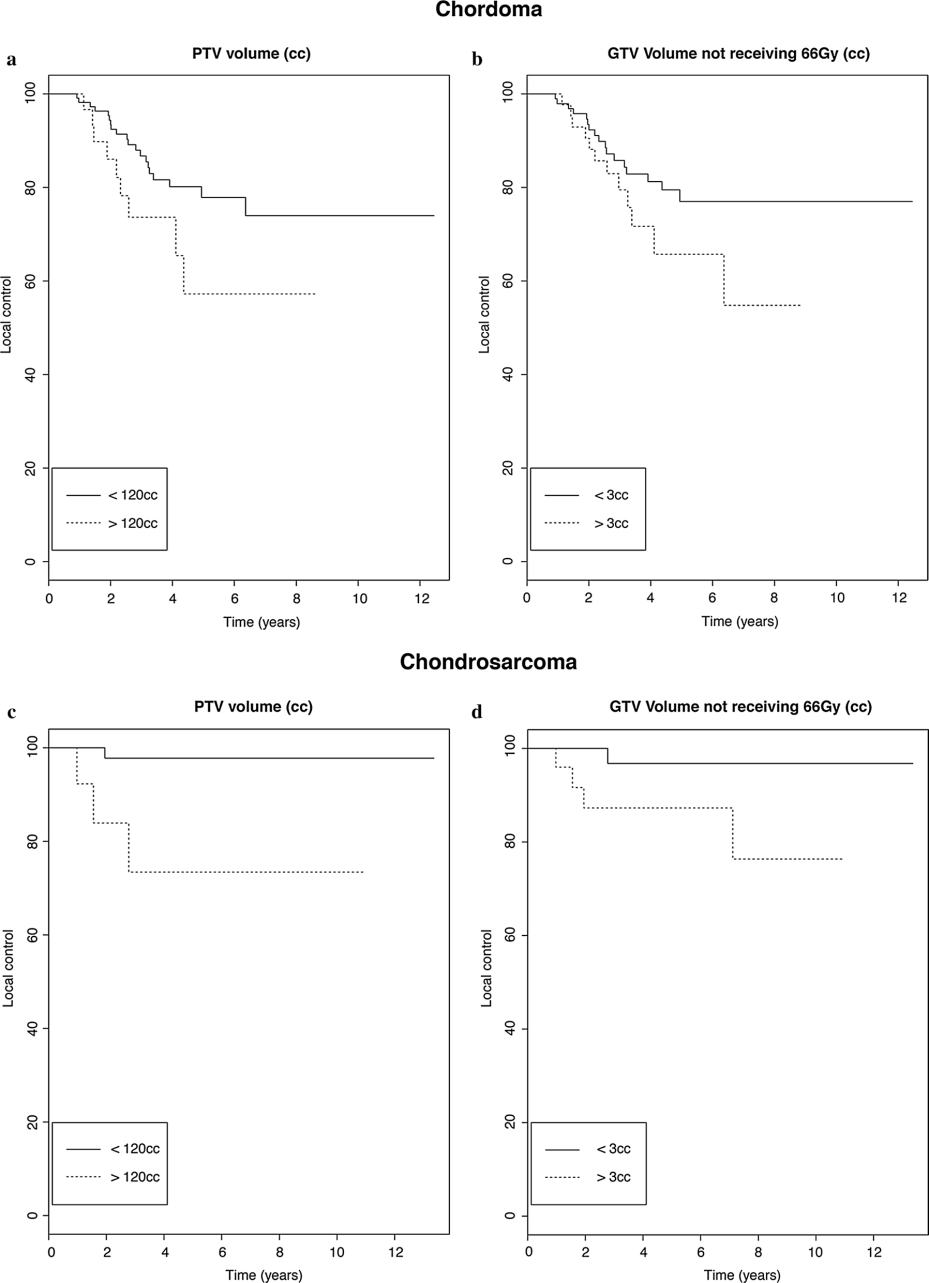
Local control in dependence of PTV_high_ volume and GTV volume receiving less than 66Gy_RBE_ separately for both chordoma and chondrosarcoma patients. Apart from tumor histology (chordoma vs chondosarcoma, *p* = 0.009), both PTV_high_ volume (*p* = 0.045) and GTV (V < 66Gy_RBE_, *p* = 0.022) were significant independent prognostic factors of local failure in the multivariate analysis. The PTV volume could differentiate between a 5-year local control rate of 57% (PTV > 120 cc) and 78% (PTV < 120 cc) for chordoma (**a**) and between 73% (PTV > 120 cc) and 97% (PTV < 120 cc) for chondrosarcoma patients (**c**). The GTV volume receiving less than 66Gy_RBE_ could differentiate between a 5-year local control rate of 65% (GTV < 66Gy_RBE_ > 3 cc) and 77% (GTV < 66Gy_RBE_ < 3 cc) for chordoma (**b**) and between 86% (GTV < 66Gy_RBE_ > 3 cc) and 97% (GTV < 66Gy_RBE_ < 3 cc) for chondrosarcoma patients (**d**)

In the multivariate analysis, histology (chordoma vs chondosarcoma, *p* = 0.009), PTV volume (*p* = 0.045) and GTV (V < 66Gy_RBE_, *p* = 0.022), (CI = 0.67) remained significant and were an independent prognostic factor for local tumor control. The GTV volume receiving less than 66Gy_RBE_ could differentiate between a 5-year local control rate of 65% (GTV < 66Gy_RBE_ > 3 cc) and 77% (GTV < 66Gy_RBE_ < 3 cc) for chordoma and between 86% (GTV < 66Gy_RBE_ > 3 cc) and 97% (GTV < 66Gy_RBE_ < 3 cc) for chondrosarcoma patients. The PTV volume could differentiate between a 5-year local control rate of 57% (PTV > 120 cc) and 78% (PTV < 120 cc) for chordoma and between 73% (PTV > 120 cc) and 97% (PTV < 120 cc) for chondrosarcoma patients. Figure 4 details the Kaplan Meier curves of local control in dependence of PTV_high_ volume and GTV volume receiving less than 66Gy_RBE_ separately for both chordoma and chondrosarcoma patients.

## Discussion

Despite combined modality treatment involving surgery and adjuvant radiotherapy, a relevant percentage of chordoma and chondrosarcoma patients develop a local recurrence [8–10]. A reason might be the close proximity or involvement/compression of critical OARs, including but not limited to the brainstem, optic chiasm and optic nerves, making complete surgical resection and the safe delivery of radiation of these tumors challenging. Unfortunately, in most cases, we currently lack prognostic parameters to assess the risk of local failure for an individual patient.

In our previous analysis, we could show that skull-base tumor patients treated with pencil-beam scanning proton therapy have a favorable outcome with a 7-year OS and LC of 80% and 78%, respectively [11]. These results compare favorably with a recently published meta-analysis about intracranial chordoma [14]. In addition, we identified optic apparatus and/or brainstem compression and histology as prognostic factors for the risk of local failure.

Another important finding was that GTV volume could be identified as an independent prognostic factor of local control, which could be confirmed in the current analysis in addition to PTV volume (Table 2). This provides a strong basis for the evaluation of a potential re-resection before PT, whenever possible, to reduce the risk of local recurrence. In a recently published meta-analysis the extent of surgical resection of 670 chordoma patients from 15 studies was analyzed [14]. The weighted mean percentage of patients receiving gross total resection was 29%, while subtotal resection was achieved in 61% and partial resection in 20% of cases. This suggests that complete resection or re-resection is not always feasible and that we might need to include additional parameters with prognostic potential to assess the risk of local recurrence and differentiate between patients with better or worse outcomes.

In this study, we could for the first time, identify prognostic DVH parameters for proton therapy delivered to chordomas and chondrosarcomas, which are associated with an increased risk of local recurrence in these patients. In our analysis, local control for chondrosarcomas was significantly better compared to chordomas, which is in line with other studies [15]. To account for the differences in radiation sensitivity of chordoma and chondrosarcoma patients, tumor histology was included as a categorial variable in the multivariate analysis and could be confirmed as an independent prognostic factor for local control. In addition, PTV volume and the GTV volume receiving less than 66Gy_RBE_ were independent prognostic factors. It should be mentioned that many of the analyzed parameters are conventional DVH parameters, and although they were not independent predictors of local failure, multiple GTV and PTV parameters were prognostic in the univariate analysis. This suggests that it might be feasible to assess the risk of local recurrence from these conventional DVH parameters, used in daily clinical routine.

Our patterns of recurrence analysis, based on 27 follow-up MRI studies of 35 patients with local recurrence, indicates that our delineation paradigm was clinically appropriate, with only one case of a surgical pathway recurrence (0.5% of total patients), which was not covered by the initial PTV. This point is highly relevant due to the absence of published guidelines for these challenging tumors. All other recurrences were either completely or at least partially covered by the initial target volumes (GTV, PTV_high_, PTV_low_). This suggests that the recurrences have developed from the initial tumor, growing into and/or infiltrating adjacent organs subsequently. The applied dose to the tumor might have been insufficient in these cases due to OAR constraints and/or the tumor cells might have been particularly radioresistant, which is however difficult to assess retrospectively (e.g. biological/molecular analysis).

In addition, the differing time intervals between the proton treatment and available follow-up imaging of the recurrences makes it difficult to compare the coverage of the recurrences by the initial target volumes. In some cases, a longer time interval might have led to an increased tumor growth outside the initial PTV or GTV.

So far, no contouring guidelines about chordomas and chondrosarcomas of the skull-base have been published, thus our analysis might provide a basis for clinical decision making and risk assessment of an individual skull-base tumor patient. Regarding target volume delineation, it should be noted that the sphenoid sinus should be covered in case of chiasm involvement, as patients with tumors in this location had a high risk of local recurrence in the sphenoid sinus in our analysis.

There are several limitations of this study, including the long time interval between the first treated patient in 1998 and the last patient of this analysis in 2012. The available imaging, imaging quality and to some extent treatment planning changed slightly over time and image registration of the recurrences can be a challenging process. Some patients were treated with single-field uniform dose (SFUD) and others with intensity modulated proton therapy (IMPT). It also needs to be mentioned that local tumor control could also be achieved in a proportion of patients, whose DVH characteristics did not fully meet the planning objectives, suggesting that individual radiation resistance may be an additional important factor. In addition, the retrospective character of our analysis is prone to bias. Strengths of our analysis include the large patient cohort, inclusion of modern IMPT techniques, as well as the high percentage of available follow-up imaging for the recurred patients.

In summary, we were able to identify prognostic DVH parameters, associated with the risk of local recurrence in chordoma and chondrosarcoma patients treated with proton therapy. We have shown that the residual tumor volume and the coverage of the PTV was of paramount importance. These metrics may be used for clinical decision making when treating these challenging patients, although confirmatory results are required.

## Data Availability

The datasets used and/or analysed during the current study are available from the corresponding author on reasonable request.

## Abbreviations

CI: Concordance index
CTV: Clinical target volume
DVH: Dose volume histogram
GTV: Gross tumor volume
IMPT: Intensity modulated proton therapy
LC: Local control
LR: Local recurrence
OAR: Organs of risk
OS: Overall survival
PBS: Pencil-beam scanning
PSI: Paul Scherrer Institute
PTV: Planning target volume
RBE: Relative biologic effectiveness
SFUD: Single-field uniform dose
